# Mice do not require auditory input for the normal development of their ultrasonic vocalizations

**DOI:** 10.1186/1471-2202-13-40

**Published:** 2012-04-25

**Authors:** Kurt Hammerschmidt, Ellen Reisinger, Katharina Westekemper, Ludwig Ehrenreich, Nicola Strenzke, Julia Fischer

**Affiliations:** 1 Cognitive Ethology Lab, German Primate Center, Kellnerweg 4, 37077 Göttingen, Germany; 2 Molecular Biology of Cochlear Neurotransmission Group, University Medical Center Göttingen, Robert-Koch-Str. 40, 37099 Göttingen, Germany; 3 Department of Otolaryngology, Auditory Systems Physiology Group, University Medical Center Göttingen, Robert-Koch-Str. 40, 37099 Göttingen, Germany; 4 Courant Research Centre 'Evolution of Social Behaviour', University of Göttingen, Kellnerweg 6, 37077 Göttingen, Germany

**Keywords:** DFNB9, Evolution, Language, Mice, Ontogeny, Otoferlin, Speech, Vocal learning

## Abstract

**Background:**

Transgenic mice have become an important tool to elucidate the genetic foundation of the human language faculty. While learning is an essential prerequisite for the acquisition of human speech, it is still a matter of debate whether auditory learning plays any role in the development of species-specific vocalizations in mice. To study the influence of auditory input on call development, we compared the occurrence and structure of ultrasonic vocalizations from deaf otoferlin-knockout mice, a model for human deafness DFNB9, to those of hearing wild-type and heterozygous littermates.

**Results:**

We found that the occurrence and structure of ultrasonic vocalizations recorded from deaf otoferlin-knockout mice and hearing wild-type and heterozygous littermates do not differ. Isolation calls from 16 deaf and 15 hearing pups show the same ontogenetic development in terms of the usage and structure of their vocalizations as their hearing conspecifics. Similarly, adult courtship 'songs' produced by 12 deaf and 16 hearing males did not differ in the latency to call, rhythm of calling or acoustic structure.

**Conclusion:**

The results indicate that auditory experience is not a prerequisite for the development of species-specific vocalizations in mice. Thus, mouse models are of only limited suitability to study the evolution of vocal learning, a crucial component in the development of human speech. Nevertheless, ultrasonic vocalizations of mice constitute a valuable readout in studies of the genetic foundations of social and communicative behavior.

## Background

Comparative analyses of the vocal behavior of nonhuman primates and humans have allowed to identify the derived features of the human language faculty, such as volitional control over vocal production, symbolic understanding, and recursion [1,2]. Moreover, screening studies have revealed the association of a series of genes with specific language disorders [3]. Advances in genetic engineering now allow the study of particular genes associated with language disorders in mouse models [4]. Mouse ultrasonic vocalizations (USVs) are the target variable in many of these studies; they also turned out to be a valuable readout in studies addressing the genetic foundation of social behavior [1,5]. The interest in mice as model for vocal learning was further spurred by the finding that adult male mice produce elaborate ultrasonic 'songs' in encounters with females, implying that learning might play a role in the ontogenetic development of these vocalizations [6]. A cross-fostering study on C57BL/6 and BALB/c mice however showed that males kept singing songs with the typical characteristics of their genetic parents, both in terms of the acoustic structure and temporal characteristics [7]. On the other hand, a developmental study on CBA/CaJ mice revealed substantial changes in their vocalizations with age [8], but it remained unclear to which extent learning is involved in these developmental changes. Clarifying this question is essential to judge the suitability of mice models for specific developmental and evolutionary, as well as clinical studies.

To shed light on the question whether mice require auditory input of their own species-specific vocalizations for a normal development of their calls, we analyzed the recordings of calls from mice that serve as a model of human deafness DFNB9. These mice have deficits in otoferlin, a synaptic vesicle protein at the cochlear inner hair cell [9]. Vesicle exocytosis is disrupted in animals lacking otoferlin, resulting in profound deafness [9,10]. We recorded pup's isolation calls before and after they reached hearing ability. Previous studies showed that neonatal mice are deaf and they only begin to hear at an age of approximately 10-12 days [11,12]. In addition, we recorded songs of deaf and normally hearing adult males during their courtship encounters. If mice fall within the pattern of most terrestrial mammals, they should not require auditory input for species-specific vocal development [13,14]. All terrestrial mammals seem to lack the ability of vocal production learning, with the exception of elephants [15]. In case mice have developed the ability of vocal production learning, we expected to find significant differences between deaf and hearing mice. In case that auditory input is essential, deaf mice should produce structurally altered calls after hearing onset.

## Results

### Pup isolation calls

**Figure 1:**
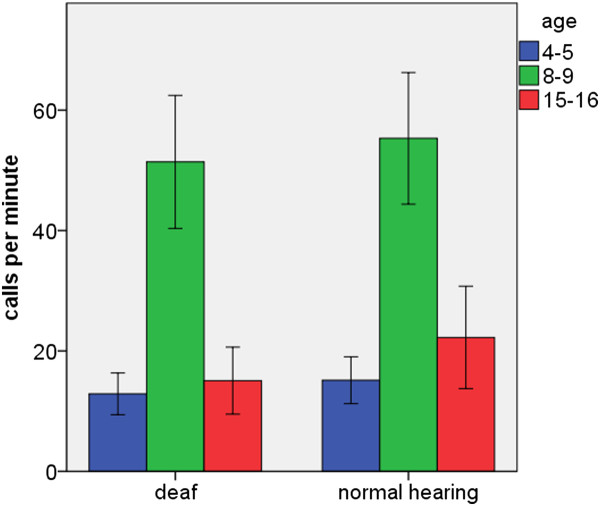
**Age related changes in pup isolation calls**. A: Number of pup isolation calls (mean ± s.e.m.).

The number of calls given by isolated pups increased from P4-5 to P8-9 and then decreased again at P15-16 (Figure 1). Accordingly, we found significant variation in relation to age (F_2,81 _= 18.1, *P *= 0.000), but no significant differences between deaf and normally hearing animals (F_1,83 _= 0.48, *P *= 0.49), and no significant interaction (F_2,81 _= 0.08, *P *= 0.93).

**Figure 2:**
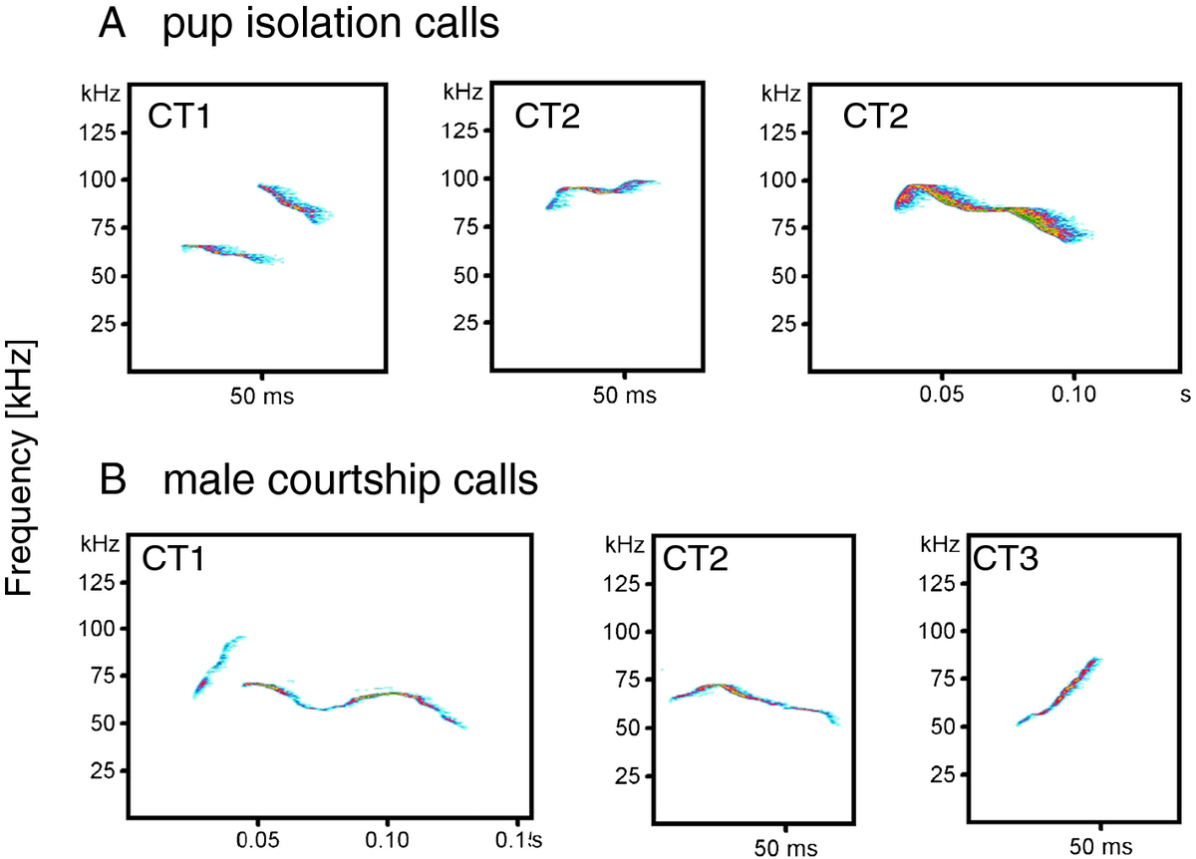
**Examples of vocal types**.

The two-step cluster analysis identified a two cluster solution as the best categorization of pup isolation calls, which is equivalent to a vocal repertoire of two (major) call types (CT). Cluster 1 (CT1) encompassed 14.1% and is defined by calls with high sudden frequency jumps (PF jump = 29.9 ± 15.9 kHz (mean ± s.d.)), high maximum peak frequency (PF max = 93.9 ± 10.6 kHz) and a long call duration (51.6 ± 19.4 ms). The second cluster (CT2 = 85.9%) consisted of calls with shorter call duration (30.3 ± 14.5 ms). It was not possible to establish reliable categories in relation to the modulation of the peak frequency, i.e. whether it is descending, flat or ascending. Exemplary spectrograms of CT1 and CT2 are presented in Figure 2A.

We next conducted a discriminant function analysis (DFA) to distinguish between calls given by *Otof *knock-out or control subjects. While the DFA assigned 96.7% of the calls to the correct call type (cross-validated = 96.5%), it was not possible to assign calls in relation to the pups' hearing ability (correct assignment = 55.8%; cross-validated = 54.8%).

The analysis of the acoustic properties of calls revealed significant age-related changes in nearly all acoustic parameters of CT2, and in a few acoustic parameters of CT1. Importantly, we found no significant differences in relation to the hearing ability of the pups or their mothers (Tables 1 and 2). Because mouse pups do not begin to hear before postnatal day 9 we conducted a separate analysis for P15-16. In line with the above results, we found no significant differences in the acoustic structure between deaf and normally hearing mice (Table 3).

**Table 1:** Description of call parameter used in the analysis

Acoustic parameters	Description
Duration [ms]	Time between onset and offset of call

Amplitude gap [ms]	Duration of breaks in amplitude within call

PF start [Hz]	Start frequency of peak frequency

PF max [Hz]	Maximum peak frequency

PF jump [Hz]	Maximum difference of peak frequency between successive bins

PF max loc	Location of PF max in relation to total call duration (1/duration) * location]

PF jump loc	Location of maximum PF jump in relation to total call duration [(1/duration) * location]

Slope of trend	Factor of linear trend of peak frequency

**Table 2:** Results of statistical tests (*P *values) of pup isolation calls in relation to hearing ability (deaf, normally hearing) and age (P4-5, P8-9, P15-16)

Acoustic parameters	Call type 1		Call type 2	
	**(14.1% of calls)**		**(85.9% of calls)**	

	**hearing**	**age**	**hearing**	**age**

Duration [ms]	0.767	0.195	0.979	**0.000**

Amplitude gap [ms]	0.708	0.248	0.979	0.661

PF start [kHz]	0.767	**0.008**	0.220	**0.000**

PF max [kHz]	0.767	0.619	0.192	**0.000**

PF max loc [rel.]	0.708	0.619	0.979	**0.000**

PF jump [kHz]	0.767	**0.012**	0.979	**0.000**

PF jump loc [rel.]	0.767	0.619	0.979	**0.000**

Slope of trend	0.859	0.326	0.979	**0.000**

Significant differences are marked bold. *P *values are corrected for multiple testing using a Simes correction

**Table 3:** Acoustic differences of mouse pup vocalizations at age P15-16 in relation to hearing ability (deaf, normally hearing)

Acoustic parameters	deaf	hearing	P values
Duration [ms]	23.8 ± 15.2	32.1 ± 18.5	0.121

Amplitude gap [ms]	0.90 ± 2.64	0.85 ± 2.96	0.915

PF start [kHz]	81.1 ± 14.0	76.5 ± 9.5	0.076

PF max [kHz]	86.6 ± 14.7	83.2 ± 10.24	0.152

PF max loc [rel.]	0.51 ± 0.32	0.49 ± 0.32	0.627

PF jump [kHz]	3.0 ± 7.0	5.1 ± 7.0	0.234

PF jump loc [rel.]	0.41 ± 0.30	0.39 ± 0.29	0.550

Slope of trend	0.08 ± 0.29	0.06 ± 0.23	0.655

Scores show mean and s.d., *P *values are not corrected for multiple testing

### Male mouse courtship vocalizations

Deaf males tended to produce a higher number of calls than normally hearing ones during their courtship encounters (mean ± s.e.m., 192.7 ± 19.1 calls/min for deaf and 119.5 ± 26.3 calls/min per minute for hearing animals; Mann-Whitney U-Test U = 55, *P *= 0.057). We found no significant differences in the latency to call (deaf: 25.6 ± 6.7 s, hearing: 20.1 ± 4.8 s; U = 78.5, *P *= 0.42) or in the mean interval duration measured from the start of the call to the start of the subsequent call (deaf: 0.30 ± 0.04 s, hearing: 0.96 ± .66 s, U = 78, *P *= 0.46).

A two-step cluster analysis identified a three cluster solution as the best possible solution. Cluster 1 (CT1) is defined by high sudden frequency jumps (PF jump: 25.3 ± 16.1 kHz (mean ± s.d.)), a high maximum peak frequency (PF max: 92.6 ± 15.8 kHz) and a long call duration (75.6 ± 36.1 ms). The second cluster (CT2) consisted of calls with a descending or flat peak frequency (according to this we found a low coefficient for maximum location of peak frequency; PF max loc = 0.22 ± 0.18), and mean call duration (47.2 ± 23.6 ms). The third call cluster (CT3) contained calls with increasing peak frequency (PF max loc = 0.80 ± 0.15) and a short call duration (25 ± 9.4 ms). Exemplary spectrograms are presented in Figure 2B. In total, 17.7% of male courtship vocalization belonged to CT1, 49.9% to CT2, and 32.4% to CT3.

**Figure 3:**
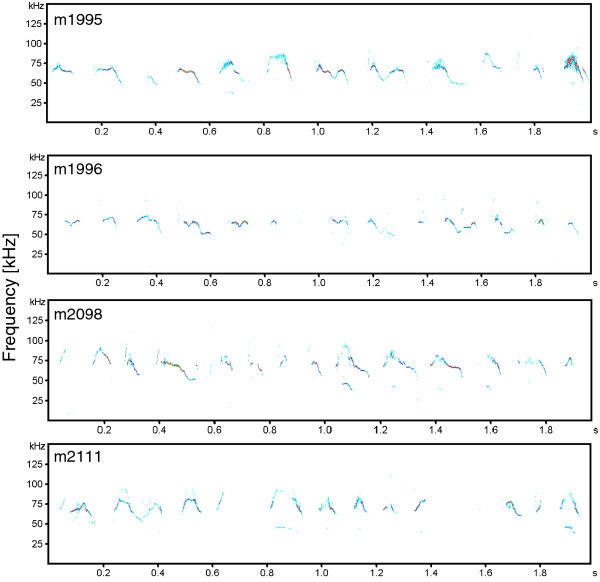
**Examples of male courtship vocalizations**. m1995 and m2111 show deaf males, m1996 and m2098 show normally hearing males.

The DFA assigned 94.3% of the calls to the correct call type (cross-validated = 93.9%), but only 54.2% of calls (cross-validated = 54.1%) to the correct category in relation to hearing ability. Along the same lines, we found no significant differences in the structure of call types between deaf and normally hearing males (Table 4 Figure 3). To ensure that the results of the statistical analyses are not simply an artifact by the chosen number of call types, we repeated the statistical analyses for different cluster solutions. The choice of a varying number of clusters did not affect the results (Additional file 1).

**Table 4:** Results of statistical tests (*P *values) of acoustic differences of male courtship vocalizations in relation to hearing ability (deaf, normally hearing)

Acoustic parameters	CT1	CT2	CT3
	**(17.7% of calls)**	**(49.9% of calls)**	**(32.4% of calls)**

Duration [ms]	0.810	0.520	0.216

Amplitude gap [ms]	0.810	0.724	0.236

PF start [kHz]	0.810	0.341	0.216

PF max [kHz]	0.810	0.362	0.542

PF jump [kHz]	0.810	0.341	0.542

PF max loc	0.640	0.914	0.542

PF jump loc	0.810	0.341	0.216

Slope of trend	0.810	0.962	0.542

*P *values are corrected for multiple testing using a Simes correction

## Discussion

We found no significant differences in calling rate or acoustic structure of mouse ultrasonic vocalizations in relation to hearing ability. This was true for pup isolations calls as well as adult male 'courtship songs'. The ontogenetic changes found in both deaf and hearing pups are similar to those reported for other hearing strains [16,17]. Apparently, auditory experience is not a prerequisite for the normal development of species-specific vocalizations in mice.

Our results are in line with findings on cross-fostered male mice courtship songs. Cross-fostered male mice did not adapt the sound characteristic of their social parents, but produced sound sequences with the acoustic structure and temporal pattern typical of their genetic parents [10]. Other studies which suggested that learning might have an influence on the development of male song structure, had demonstrated developmental changes [8] and a high complexity of male songs [6]. However, neither criterion is a demonstration of learning per se. Alternatively, developmental changes may be attributed to maturation, while complexity could be an outcome of selective pressures or simply be a by-product of nonlinear phenomena in sound production [18].

While we found no differences in relation to hearing ability within our mouse strain, there is now evidence accumulating that genetic differences between mouse strains may affect calling rate, call duration and frequency characteristics of isolation calls [17,19]. Mice homozygous for non-functional FoxP2 alleles produced significantly fewer isolation calls than their wild-type littermates, whereas heterozygous mouse pups produced nearly the same amount of vocalizations as their wild-type littermates [20,21]. However, homozygous mouse pups exhibit severe developmental deficits and die around 3 weeks after birth, implying that the reduction in ultrasonic vocalization might not represent specific effects of FoxP2 on these vocalizations [20]. In addition heterozygous mouse pups did not differ significantly from wild-types in the structure of their ultrasounds [20,21], implying that functional FoxP2 alleles are not important for the production of species-specific ultrasounds as long as a normal development is guaranteed. In addition, androgenic hormones can have a profound influence on male courtship vocalizations [5,22]. Further, it was shown that different kinds of female odors lead to significant changes in the structure of courtship vocalization [23,24]. In sum, both genetic and environmental features may alter the occurrence and structure of mouse USVs, but auditory input does not appear to play a substantial role. It seems promising though to study the influence of hearing on the development of social behavior. Vocalizations have an important function in regulating the social relationships between animals, and it seems probable that deaf mice develop disrupted social relationships.

A critical issue of studies revealing no differences between categories is the question whether the used method is adequate to reveal possible differences. The discriminant function analysis has a long history in bioacoustics research and had regular shown that this procedure is able to detect subtle differences. A recent example is a study on crested gibbons where the authors found subtle differences in songs of close related populations, which are not detectable by ear [25]. A second study on chiffchaff calls is a good example because the call structure of these calls is comparable to the whistle like structure of mouse ultrasounds. The discriminant function analyses revealed the individual signature as well as micro-geographical variation among the different recording sites [26]. In both cases the correct assignment was above 90% with chance levels around 5%. In our study the correct assignment to deaf and normal hearing mice was app. 55% and a change level of 50%. There are many other studies showing that a discriminant function analysis is a powerful tool to reveal existing differences. A further critical issue of the present study may be the low number of call categories revealed by the acoustic analysis. This number, however, was based on a reproducible procedure which determines the optimal number of clusters by comparing different solutions using the Bayesian Information Criterion (BIC). Moreover, using cluster solutions with higher number of categories did not affect the results (Additional file 1). Notably, increasing the number of clusters leads to the occurrence of call types that are produced by one individual only. In other words, apart from the general call types identified with the current procedure, the remaining variation is largely due to individual differences but not to sub-categories that can be found across individuals.

Taken together, our results suggest that neither exposure to auditory input from others, nor auditory feedback of the subject's own vocalizations is important for the development of species-specific vocalizations. Auditory experience with the species-specific vocalizations as well as auditory feed-back was shown to be important in human speech [1] and bird song [27], both learned modes of production. Therefore, it is questionable whether mice constitute a suitable model to study the genetic foundation of vocal learning.

## Conclusions

Deaf mice revealed the same ontogenetic development in terms of the number, usage and structure of their pup vocalizations as their hearing conspecifics. Similarly, there were no differences in male courtship songs in relation to hearing ability. Apparently, mice do not require auditory input for normal vocal development. These findings question the utility of mouse models to elucidate the foundations of vocal learning, a key component in the development of human speech. Nevertheless, ultrasonic vocalizations of mice constitute a valuable readout in studies of the genetic foundations of social and communicative behavior, such as autism spectrum disorders [19,28].

## Methods

Otoferlin knock-out animals were generated as described in [29]. We analyzed the vocal behavior from 16 *Otof *knock-out (*Otof*^-/-^, deaf) and 15 heterozygous (*Otof*^+/-^, hearing) pups at postnatal age of four or five days (P4-5), eight or nine days (P8-9) and 15 or 16 days (P15-16) while isolated from their mothers. Pups were bred using *Otof*^-/- ^females with *Otof*^+/- ^males and thus raised by deaf females. All mice were of mixed background (129 ola and C57N). Litters and mothers were kept in standard cages, one litter per cage. For identification, pups were marked with tattoos on the paws one day after birth.

In addition, we analyzed the vocal behavior from 12 knock-out and 16 control males. For the adults, deaf mice (*Otof*^-/-^) and heterozygous *Otof*^+/- ^mice of 129ola/C57N mixed background were raised in groups of 2 to 5 males per cage until they were 8-9 weeks old. Each male was put to a separate cage in which the courtship songs were recorded one day after isolation. For the recordings, their cages were placed in a sound-attenuated Styrofoam box. After three minutes, a female was introduced for three minutes.

All experiments complied with national animal care guidelines and were approved by the University of Göttingen Board for animal welfare and the animal welfareoffice of the state of Lower Saxony (AZ 33.11.42502-04-044/08).

### Acoustic Recordings

Mouse pups were recorded three times at postnatal age of four or five (P4-5), eight or nine (P8-9) and 15 or 16 days (P15-16). For each recording the cage with the litter to be measured was taken to a bench (room temperature: 21-22 C) and pups were selected randomly, weighed and placed in a soundproofed custom made plastic box (diameter 13.5 cm). An ultrasound microphone (UltraSoundGate CM16) was placed in the lid of the box 12 cm above the bottom and connected to a preamplifier (UltraSoundGate 116) which was connected to a notebook computer. We recorded the pups for 150 seconds using the recording software Avisoft Recorder 3.4 with a sampling frequency of 300 kHz (hardware and software from Avisoft Bioacoustics, Berlin, Germany). In older pups, short-term isolation from mother did not evoke isolation calls in a predictable manner. Therefore we put for the P15-16 pups with the plastic box in ice water (bottom temperature = 5-6 C), and recorded for 180 seconds. We chose such a relatively old age to record the pups because they do not start to hear before day 10-12 [12].

To elicit courtship songs from male mice, we isolated males at the age of two months one day before the recording in a macrolon 2 cage (36.5 × 21 × 14 cm). On the recording day we placed the males in their own cage in a sound-attenuated styrofoam box (30 × 43 × 24 cm). After three minutes we introduced a female for three minutes and recorded the vocalizations with the same recording equipment we used for the recording of the pup isolation calls. The ultrasound microphone was placed in the lid of the box, 24 cm above the floor.

### Acoustic analysis

We counted the number of calls per recording session with AVISOFT Recorder 3.4. To separate isolation calls from the rest of the recording we used the whistles detection algorithm with following selection criteria: possible changes per step = 4 (4687 Hz), minimal continuity = 8 ms, possible frequency range = 40 to 150 kHz. These criteria were compared with former analysis of pup vocalizations [4]. In addition, we visually controlled the procedure to ensure that the automated sampling routine selected only calls of mouse pups and no other sounds such as toe clicking. The AVISOFT recorder software stores the selected sounds in separate wave files, and, in addition logs the time of call onset.

From the stored calls, we calculated spectrograms (frequency range: 150 kHz, frequency resolution: 293 Hz, time resolution: 0.21 ms). We submitted the resulting spectrograms to the custom software program LMA 2011 to extract a set of characteristic acoustic parameters. As mice typically concentrate the energy of their USV in a single-frequency band, so-called 'pure tone-like sounds' or 'whistles', we focused on peak frequency of USV, i.e. the loudest frequency of a respective time frame. Just small head movements can lead to strong amplitude fluctuations in USVs. In addition, mice produce often soft sounds in the ultrasonic range. To ensure correct parameter estimations, we visually controlled the estimation and excluded incorrect estimated calls from the analysis. For each call we determined the duration of a call and the duration of amplitude gaps within a call (sound parts whose intensity is below 10% (at the start) and 15% (at the end of call) of the mean maximum amplitude of a call). Furthermore, we determined start, maximum peak frequency, as well as the greatest difference in peak frequency between two consecutive 0.21 ms bins. Typical whistles concentrate their energy to one amplitude peak. Therefore, the peak frequency corresponds to the fundamental frequency, although it is difficult to prove as long as no harmonics can be detected. In addition, we calculated the location of the maximum frequency and the location of peak frequency jump within the call. To describe the call modulation we calculated the slope of a linear trend through the peak frequencies of consecutive 0.21 ms bins. We did the same calculation for the male vocalizations (Table 1). In addition to estimating the number of given calls, we estimated the latency to call.

### Recording of auditory brainstem response (ABR)

**Figure 4:**
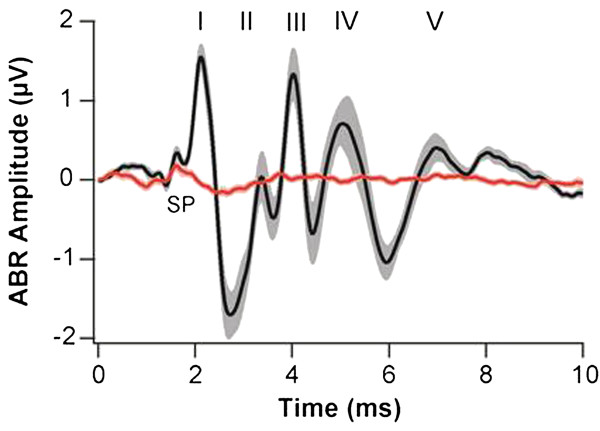
**ABR waveforms**. Grand averages ± s.e.m. of ABR waveforms in response to 100 dB click stimuli presented at 20 Hz in 15 Otoferlin knockout animals (red) and 14 heterozygous littermates (black) used for pup vocalization studies. Roman numbers denote ABR wave peaks I-V according to Jewett in wild type animals, SP: putative summating potential component observed in knockout and control.

To confirm correct genotyping and exclude hearing impairment in control animals, auditory brainstem responses (ABR) to click stimuli were recorded from 57 out of 59 animals in the analysis. Measurements in heterozygous controls yielded typical ABR waveforms with 5 waves representing synchronous postsynaptic potential generation in the auditory nerve (wave I) and brainstem with a mean threshold of 32 ± 2 dB peak equivalent (Figure 4). Otoferlin knockout animals only showed a small early wave component at high sound intensities (mean threshold 100 ± 2 dB), which most likely represents the summating potential, reflecting normal hair cell transduction currents upstream of the synaptic deficit. In summary, ABR recordings confirmed normally hearing in all control animals and were consistent with profound deafness due to abolished hair cell exocytosis in otoferlin knockout animals.

Animals were anaesthetized intraperitoneally with a combination of ketamine (125 mg kg-1) and xylazine (2.5 mg kg-1) and the heart rate was constantly monitored to control the depth of anaesthesia. The core temperature was maintained constant at 37°C using a rectal temperature-controlled heat blanket (Hugo Sachs Elektronik - Harvard Apparatus GmbH, March-Hugstetten, Germany). For stimulus generation, presentation and data acquisition we used the TDT III Systems (Tucker-Davis-Technologies, Ft Lauderdale, FL) run by custom-written Matlab software (The Mathworks). Clicks of 0.03 ms duration were calibrated using a ¼″ Brüel and Kjaer microphone (D 4039, Brüel & Kjaer GmbH, Bremen, Germany) and were presented at 20 Hz in the free field ipsilaterally using a JBL 2402 speaker (JBL GmbH & Co., Neuhofen, Germany). The difference potential between vertex and mastoid subdermal needles was amplified 20 times and sampled at a rate of 50 kHz for 20 ms, 2 × 2000 times to obtain two mean ABRs for each sound intensity. Hearing threshold was determined with 10 dB precision as the lowest stimulus intensity that evoked a reproducible response waveform in both traces by visual inspection.

### Statistics

We used a two-step cluster analysis (CA, SPSS 19) to establish vocal categories. We used the log-likelihood distance measure to establish different vocal cluster (up to 15 clusters) and the Schwarz-Bayesian information criterion (BIC) to decide which cluster solution showed the best fit. We used the eight acoustic parameters described above to calculate the CA. A higher number of parameters would have provided no advantage, because highly correlating acoustic parameters render it difficult to find appropriate cluster centers. In addition, they will shift the result in the direction of the most highly correlating parameters. The acoustic analysis program provided a set of further parameters. However, these parameters obtained a high correlation with the already chosen parameters (correlation coefficient above 0.7). Therefore, there was no advantage to include theses parameters in the analysis. They only would lead to an increase to correct for multiple testing. To confirm the cluster solution and to estimate the contribution of different acoustic parameters to distinguish between the established call categories we conducted a discriminant function analysis (DFA, SPSS 19) with the same eight acoustic parameters. We used a stepwise DFA. The selection criterion for an acoustic parameter to be entered was p = 0.05 and p = 0.1 to be removed from the analysis. The assignment of the calls was cross-validated by the leaving-one-out method of SPSS 19.

To ensure that the statistic results are not simply an artifact by the chosen number of call categories, we calculated for the male mouse courtship analysis call type solution of higher order and tested them for differences in relation to hearing ability (Additional file 1).

To test for differences in structure and number of isolation calls between deaf and normally hearing pups we used a mixed linear model (SPSS 19) with hearing ability, recording age (P5-6, P8-9 and P15-16) as fixed factors, and subject, weight and litter as random factor. To test the courtship vocalization for structural differences regarding hearing ability we used a mixed linear model (SPSS 19), with hearing ability as fixed factors and subject as random factor. We conducted separate tests for all vocal types. To test the courtship vocalization for differences regarding call number, latency to call, rhythm of calling (start/start intervals) and call type usage we used Mann-Whitney-U test (SPSS 19). Where it was necessary we applied a Simes correction to correct for multiple testing. We chose Simes correction because it belongs to the correction methods which minimize the β error.

## Competing interests

The authors declare that they have no competing interests.

## Authors' contributions

KH and JF designed the study, KH, LE and KW recorded the data, ER provided the animals and conducted the experiments, NS conducted the auditory brainstem response, and KH, NS and JF wrote the paper. All authors read and approved the final manuscript.

## Supplementary Material

Additional file 1 **Significant differences (*P *values) in call type usage of deaf and normally hearing mice in relation to number of call types**. Click here for file
