# Supplementary material for: Mice do not require auditory input for the normal development of their ultrasonic vocalizations
Source: BMC Neurosci. 2012 Apr 25;13:40. doi: 10.1186/1471-2202-13-40 (PMC3350408; doi:10.1186/1471-2202-13-40)
Supplement: Additional file 1 — Significant differences (P values) in call type usage of deaf and normally hearing mice in relation to number of call types. [file 1471-2202-13-40-S1.DOC]

Additional File 1: Significant differences (*P* values) in call type usage of deaf and normally hearing mice in relation to number of call types.

|  | **Number of call types** | | | |
| --- | --- | --- | --- | --- |
| **Call types** | **3 call types** | **5 call types** | **7 call types** | **9 call types** |
| ct1 | 0.233 | 0.733 | 0.865 | 0.813 |
| ct2 | 0.974 | 0.068 | 0.04 | 0.889 |
| ct3 | 0.843 | 0.973 | 0.973 | 0.137 |
| ct4 |  | 0.766 | 0.667 | 0.842 |
| ct5 |  | 0.741 | 0.332 | 0.894 |
| ct6 |  |  | 0.319 | 0.288 |
| ct7 |  |  | 0.305 | 0.217 |
| ct8 |  |  |  | 0.288 |
| ct9 |  |  |  | 0.409 |

We did not correct for multiple testing to get a better illustration of the lack of influence.
